# Nanofibers based on zein protein loaded with tungsten oxide for cancer therapy: fabrication, characterization and in vitro evaluation

**DOI:** 10.1038/s41598-023-49190-2

**Published:** 2023-12-14

**Authors:** Gomaa El Fawal, Ashraf M. Omar, Marwa M. Abu-Serie

**Affiliations:** 1 Polymer Materials Research Department, Advanced Technology and New Materials Research Institute (ATNMRI), City of Scientific Research and Technological Applications (SRTA-City), New Borg El-Arab City, Alexandria 21934 Egypt; 2 Medical Biotechnology Department, Genetic Engineering and Biotechnology Research Institute, City of Scientific Research and Technological Applications (SRTA-City), New Borg EL-Arab City, Alexandria 21934 Egypt

**Keywords:** Biotechnology, Cancer, Materials science

## Abstract

Plant proteins have become attractive for biomedical applications such as wound dressing and drug delivery. In this research, nanofibers from pristine zein (plant protein) and zein loaded with tungsten oxide (WO_3_) were prepared (WO_3_@zein) using less toxic solvents (ethanol and acetic acid). Morphological and biological properties of the zein nanofiber were determined. Prepared nanofibers were defined by thermogravimetric analysis (TGA), X-ray diffraction (X-RD), Fourier-transform infrared spectroscopy (FT-IR), and scanning electron microscopy. The average fiber diameter was unchanged with an increase in WO_3_ concentration from 0.001 to 0.008%. FT-IR spectroscopy and X-RD indicated the presence of WO_3_ in WO_3_@zein nanofibers. In comparison to WO_3_-free, WO_3_@zein nanofibers showed higher safety and preserved the anticancer effect of WO_3_ against human melanoma cell line (A375) melanoma cells compared to WO_3_-free. Moreover, both WO_3_-free and WO_3_@zein caused a fourfold increase in the cellular proliferation of reactive oxygen species (ROS) in the treated A375 cells compared to untreated cells. ROS elevation led to apoptosis-dependent cell death of A375 cells as evidenced by up-regulating the expression of p53-downstream genes (p21 and Bax) (tumor-suppressor gene) while down-regulating the expression of key oncogenes (BCL2 and cyclin D). In conclusion, the prepared nanofiber represents a promising and safe candidate for anticancer applications.

## Introduction

Cancer is currently recognized as a global problem that unfortunately lacks a worldwide solution^1^. Predestined annual cancer cases were 18.1 million including 9.6 million deaths-2 years ago- which is expected to double by 2040^2^. Radiation therapy, chemotherapy, and surgery are used as anticancer therapies, but they all have cons such as significant side effects and limited effectiveness^3^. Cancer issue awareness pushes the scientific community to embark on extensive mechanistic studies and different drug discovery protocols^4^. The scientific community is currently interested in nanoparticles (NPs) because of their applicable properties and distinctive structural properties^5^. NPs were used to target cancer tissue precisely due to their small size^5^. It inducts reactive oxygen species (ROS) that prompt apoptotic cell death in cancer^6^. ROS play a critical role in both cell cycle progression and apoptosis^6^. Metal oxides (inorganic NPs) are promising materials for biomedical applications (such as cancer therapy, drug delivery, and cell imaging)^7,8^. For example, cobalt, iron, and nickel NPs are used in medical biotechnology due to their magnetic properties^9^. Metal oxide NPs were used as a potential therapeutic against cancer since they caused many effects such as DNA damage, genotoxic effects, induction of oxidative stress, and anti-inflammatory responses^10^. Tungsten oxide (WO_3_) NPs is a fascinating transition metal oxide with inclusive applications such as electrochromic, sensors, anti-cancer, and antimicrobial due to its unique structures and properties^11–13^. There are various methods to prepare WO_3_ NPs with diverse morphologies such as nanofibers, nanosheets, one-dimensional nanoneedles, or two-dimensional nanoplates^14–16^. NPs can be incorporated into polymer nanofibers via electrospinning polymer solutions containing NPs^16,17^. For instance, a facile one-step electrospinning technique was used to construct mesoporous PtO − WO_3_ nanofibers for highly sensitive and selective acetone sensing^17^.

In recent years, there has been an incredible surge in the demand for polymeric nanofibers. These remarkable materials hold great promise in a wide range of applications, such as tissue engineering, blood vessels, the nervous system, drug delivery, protective clothing, filtration, and sensors^18–20^. Nanofibers were prepared by different methods such as drawing techniques, the spinneret-based tunable engineered parameter (STEP) method, phase separation, self-assembly, template synthesis, freeze-drying synthesis, and interfacial polymerization^21^. The electrospinning technique has recently attracted attention because of its unusual properties^18,22^. Notable progress has been made in engineering of electrospun nanofibers and the development of electrospinning techniques to facilitate several applications. Electrospinning produces fibers with tens of nanometer diameters^18^. Various polymers (natural and synthetic) are used for preparing nanofibers via electrospinning which have tremendous potential for biomedical applications^18^. Synthetic polymers are limited for medical applications due to their non-degradable nature, the diversity of chemical composition, or complications owing to their degradation products^20^. While natural polymers display many vital features, including biocompatibility, biological stability, and biodegradability; thus, they are superior to synthetic polymers^19,21^. Zein protein -an example of natural polymers- is categorized as a safe material by food and drug administration (US-FDA)^23^. Also, it is one of the plant proteins that is used in tissue engineering, food industry, and medical applications^24^. It is commonly used as a carrier for controlling the release of hydrophobic drugs due to its hydrophobic properties^23^. Recently, zein microsphere containing quercetin has been expanded as a promising scaffold for tissue engineering^25^. Zaersabet et al. research has shown that 3D zein scaffold could be a potential candidate for bone tissue engineering due to their promising surface topography, osteoinductivity, biodegradability, and mechanical behavior^26^. Several herbs and anticancer drugs such as curcumin, exemestane/resveratrol, and daidzin were encapsulated in zein nanofiber^27–29^. Although the formation of nanofibers from the huge majority of protein polymers is slightly difficult – because of their complicated macromolecular structure- zein can simply form fibers^30^. Zein scaffolds were found to have a high porous-walled structure, porosity, microbial attack resistance, adequate biocompatibility, and antioxidant activity^26,31^. But, the morphological stability and mechanical weakness of zein fibers in wet conditions are the key limitations. To tackle this issue, it was found that using chemical cross-linkers such as citric acid or glutaraldehyde or incorporating other synthetic polymers enhanced the mechanical properties of prepared zein fibers^32–34^. For instance, cross-linked zein fibers (using citric acid) could retain their fibrous structure after submersion in phosphate buffer saline (PBS) for two weeks at 37 C^34^. Also, addition of polyvinyl alcohol (PVA) and gelatin to zein electrospun (PVA/Zein/Gelatin) has been fabricated to enhance the shelf life of food by assuring the food quality^35^.

In this study, we employed the electrospinning technique to create polymeric scaffolds using a natural polymer called zein protein. These scaffolds were loaded with Tungsten oxide, resulting in the development of a unique material scaffold (WO_3_@zein) that exhibits enhanced characterization suitable for biomedical applications. Nanofibers scaffolds were characterized by Scanning Electron Microscopic Images (SEM), Fourier transform infrared spectroscopy (FT-IR), X-ray diffraction (X-RD), and thermal analysis (TGA). MTT assays on normal human skin melanocyte (HBF4) cell line were performed to assess the cytotoxicity. Finally, the anticancer effect of the WO_3_@zein nanofiber was evaluated against A375 human melanoma cell line. These nanofibers scaffold could be a promising candidate for treating melanoma

## Experimental

### Materials

Zein protein was purchased from Sigma-Aldrich (St. Louis, MO, USA). Ethanol (95%) and acetic acid (96%) were purchased from Lonza (New Jersey, USA). Dimethyl sulfoxide (DMSO) was purchased from Merck (Germany). Dulbecco’s Modified Eagle’s Medium (DMEM) was purchased from Lonza (USA), while fetal bovine serum (FBS) was purchased from GIBCO Company (USA). 3-(4,5-Dimethylthiazol-2-yl)-2,5-diphenyltetrazolium bromide (MTT) was purchased from Sigma Aldrich (Germany). SYBR-green PCR assay kit, cDNA synthesis kit, and Gene JET RNA purification kit were purchased from Thermo Fisher Scientific (Waltham, Massachusetts, USA). ﻿Tungsten oxide was provided by our colleague in our institute as it was prepared as earlier reported^36^. Human melanoma cell line (A375) was provided kindly from Genetic Engineering and Biotechnology Research Institute, City of Scientific Research and Technological Applications (SRTA-City), New Borg EL-Arab City, 21934, Alexandria, Egypt. All chemicals had high purity and were used without any additional purification.

## Methods

### Preparation of WO_3_@Zein nanofiber

The zein (40% w/v) was dissolved in acetic acid (96%) and ethanol (95%) in a ratio of 1:1 and stirred for 1 h. To incorporate tungsten oxide in zein nanofiber (WO_3_@zein), WO_3_ in amounts of (0.001 and 0.008%) was dissolved in zein solution just before electrospinning to get WO_3_@zein1 and WO_3_@zein8, respectively (Table 1). The formed zein and WO_3_@Zein solutions were transferred to a polypropylene plastic syringe with a 20G stainless steel blunt needle. Afterwards, the net zein solution was electrospun at a voltage of 17 kV from a high voltage source (nanoNC, electrospinning system, Model: ESR100D, Seoul, Korea). The nanofibers were collected on a static plate collector at 20 cm from the needle, keeping the feeding rate at 0.25 mL/h. While, WO_3_@zein solution was electrospun at a voltage of 15 kV, 0.3 mL/h (feeding rate) and 20 cm (collector distance). The nanofibers were collected on sheets of aluminum foil, dried under vacuum overnight to eliminate the residual acetic/ethanol solvent. They kept in a dry/cold condition to prevent any possible contamination. Neat zein and WO_3_@zein nanofibers scaffold were cross-linked using glutaraldehyde vapor. Table 1 describes nanofibers scaffolds designation and WO_3_ concentration.

**Table 1:** Concentration of WO_3_ in WO_3_@zein nanofiber scaffold.

WO_3_ concentration (%)	Designation of nanofiber scaffold
0	Neat zein
0.001%	WO_3_@zein1
0.008%	WO_3_@zein8

### Characterization of WO3@zein nanofiber

#### Scanning electron microscopic images

The morphology of the nanofibers scaffold was assessed by SEM analysis (JEOL, JSM-6460LV, Tokyo, Japan). Before SEM analysis, the samples were sputtered with gold using a sputter coater (JOEL, Tokyo, Japan). Average fiber diameters were calculated from measurements of 50 fibers from each sample using Image J software (National Institute of Health, Bethesda, MD, USA).

#### Fourier transform infrared spectroscopy (FT-IR) analysis

ATR FT-IR-8400 S (Shimadzu, Kyoto, Japan) was used to record IR spectra of the WO_3_@zein nanofibers scaffolds. For all spectra, thirty scans were collected from 4000 to 400 cm^−1^ wavelength with a 4 cm^−1^ resolution.

#### X-ray diffraction (X-RD) analysis

X-ray diffraction analysis of net zein and WO_3_@zein nanofibers scaffolds was carried out using Shimadzu X-Ray diffraction 7000 (Shimadzu, California, USA). The radiation wavelength was 1.5406. The data were acquired in the form of 2θ versus intensity (a.u) chart.

#### Thermogravimetric analysis (TGA)

Thermogravimetric Analyzer TGA-50 (Shimadzu, Kyoto, Japan) was used to study thermal stability for prepared nanofibers scaffolds under a nitrogen atmosphere (10 ml/min). Temperature scale was set from 35 to 700 °C with a heating rate of 20 °C/min.

### Cytotoxicity evaluation for WO3@Zein nanofiber

Cytotoxicity of WO_3_-free and WO_3_@zein nanofibers scaffolds was evaluated against a normal human skin melanocyte (HBF4) cell line. DMEM medium (supplemented with 10% FBS) was used to culture HBF4 in a 96-well cell culture plate (5 × 10^3^ cells per well) and incubated at 37 ºC in a 5% CO_2_ incubator. After 24 h for cell attachment, the nanofibers scaffolds WO_3_@zein as well as WO_3_-free (0.3, 4.3, and 8.6 mM), were incubated (separately) with HBF4 cells for 72 h (as previously reported^37^). The cell viability was then evaluated by MTT method^38^. Effective safe concentration (EC_100_) of WO_3_-free and WO_3_@zein nanofibers scaffolds at which cell viability is 100% was estimated by the Graphpad Instat software. Additionally, morphological alterations of treated HBF4 relative to the untreated cells were captured using a digital camera fitted on a phase contrast inverted microscope (Olympus, Japan).

### Anticancer activity evaluation of WO3@Zein nanofibers scaffolds

The anticancer effect of the WO_3_@zein nanofibers scaffolds was evaluated against A375 human melanoma cell line cultured in DMEM (supplemented with 10% FBS) and seeded in sterile 96-well plates (4 × 10^3^ cells/well). After 24 h, the nanofibers scaffolds WO_3_@Zein as well as WO_3_-free (0.3, 4.3, and 8.6 mM) were incubated with A375 cells for 72 h at 37ºC in a 5% CO_2_ incubator. The percentage of growth inhibition of A375 cells was calculated at each corresponding dose, relative to the untreated cells using the MTT method. Moreover, cellular morphological changes before and after treatment with the most effective and safest concentration of WO_3_@Zein nanofibers scaffolds were captured using a phase contrast inverted microscope with a digital camera (Olympus, Japan).

### Measurement of the increment in intracellular ROS generation in A375 cells

The cellular ROS level was quantified after incubation of the untreated and treated A375 with 2′,7′-dichlorodihydrofluorescein diacetate (DCFH-DA) for 48 h. DCFDA is oxidized by cellular ROS to fluorescent DCF, which was assessed using a spectrofluorometer (BMG Labtech, Germany) at 480 nm excitation and 530 nm emission. The relative fold change in ROS content in the treated cells relative to the untreated was calculated.

### Quantitative detection for the change in the expression of proapoptotic genes and oncogenes in the treated cancer cells

Total RNAs of untreated and the tested anticancer compounds-treated A375 cells were extracted using Gene JET RNA Purification Kit (Thermo Scientific, USA). The cDNA was synthesized from mRNA using cDNA Synthesis Kit (Thermo Scientific, USA). Real-time PCR was performed using SYBR green master mix and specific primers (Forward/Reverse) were mentioned in Table 2. The change in gene expressions in the treated cancer cells, relative to the untreated cancer cells, was estimated using the 2^−ΔΔCT^ equation (it is a simple formula used to calculate the relative fold gene expression of samples when performing real-time polymerase chain reaction).

**Table 2:** Primer sequences (forward/reverse) of the tested genes.

Gene	Primer sequences (forward/reverse)
p21	5′-CTGGGGATGTCCGTCAGAAC-3′/5′-GCCATTAGCGCATCACAGT-3′
BAX	5′-CCGCCGTGGACACAGAC-3′/5′-CAGAAAACATGTCAGCTGCCA-3′
BCL2	5′-CTGGTGGACAACATCGCCCT-3′/5′-TCTTCAGAGACAGCCAGGAGAAAT-3′
Cyclin D	5′-TACTCTGGCGCAGAAATTAGGTC-3′/5′-CTGTCTCGGAGCTCGTCTATTTG-3′

### Statistical analysis

The data are expressed as mean ± standard error of the mean (SEM) and the significant values were considered at *p* < 0.05. The unpaired T-tests were used for evaluating the difference between the mean values of the studied treatments. The analysis was done for three measurements using SPSS software version 16.

## Results and discussion

### Characterization

#### Scanning Electron Microscopic Images (SEM) for nanofibers scaffolds

**Figure 1:**
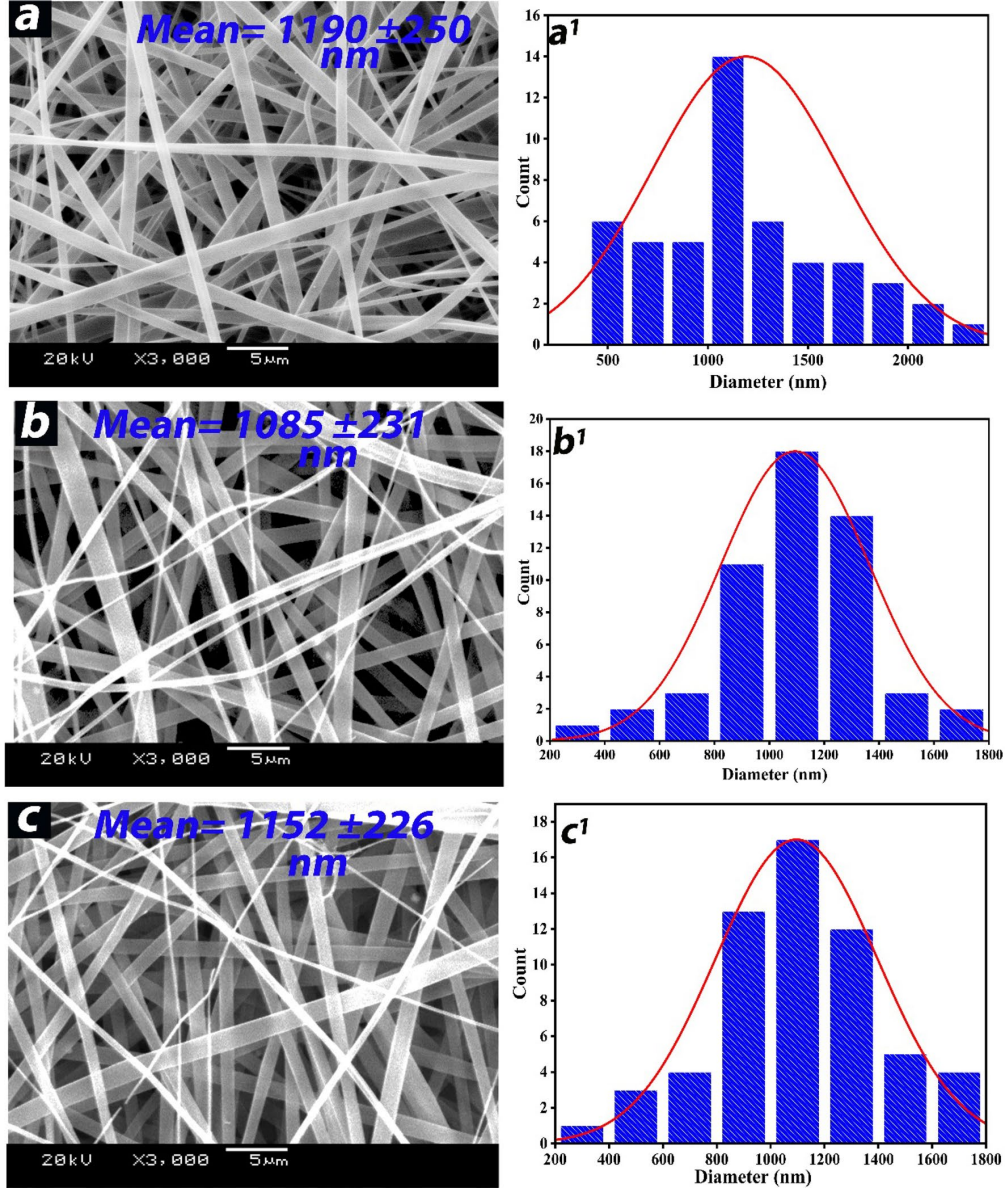
SEM images of the nanofiber (**a**) neat zein; (**b**) WO_3_@zein1; (**c**) WO_3_@zein8; a^1^, b^1^, c^1^ are nanofibers distribution of a, b, and c images respectively (Where zein (40%), tungsten (WO_3_) concentration- is 0.001% (WO_3_@zein1) and 0.008% (WO_3_@zein8)).

Figure 1 shows SEM images of neat zein and WO_3_@zein nanofibers scaffolds that show a ribbon-like morphology with smooth fibers. The average fiber diameter of neat zein nanofiber scaffold (1190 nm) is insignificantly different from WO_3_@zein1 (1085 nm) and WO_3_@zein8 nanofiber (1152 nm). Ribbon-like morphology was also reported by Selling et al. when zein was dissolved in aqueous ethanol^39^. Also, our SEM results agree well with earlier reported results when zein was used as carriers to stabilize (*-*)-epigallocatechin gallate^40^. Ribbon-like morphology could be created by the formation of “garden hose” structures that fall to form ribbon-like as they land on the collector^41^. “Garden hose” structure formed due to the solution droplets (during the electrospinning step) is observed to have an outside skin which collapsed inner as the solvent evaporated^41^. Morphology and diameter of electrospun zein are affected by several parameters, such as solvent, polymer concentration, distance between collector and spinneret, solution flow rate, and applied DC voltage^42^. For example, when we used a low concentration of zein (< 40%), beads and fibers were formed and when we used a high concentration (40%), we got continuous fibers. Increasing concentration causes molecular chain entanglement, which prevents the polymer jet from breaking up into droplets^40^. Also, increasing polymer concentration (to a specific limit) boosts the interaction between the solvent and the polymer, thus reducing the tendency of the polymer solution to form droplets under the effect of solvent surface tension^40^.

#### Fourier transform infrared spectroscopy (FT-IR) for nanofibers scaffolds

**Figure 2:**
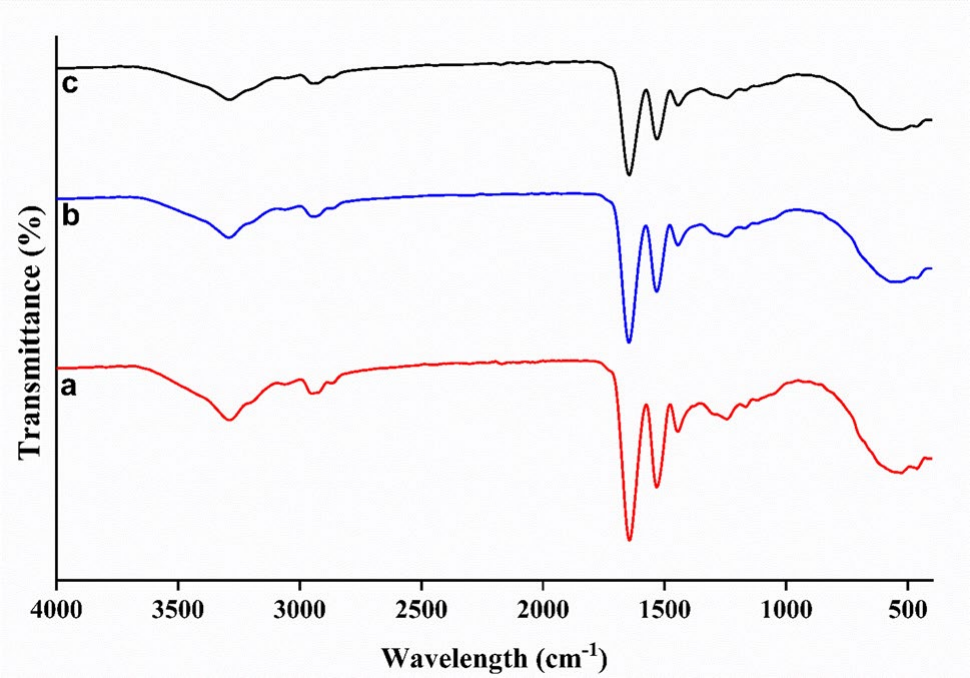
FT-IR spectrum of the nanofiber (**a**) neat zein; (**b**) WO_3_@zein1; (**c**) WO_3_@zein8; Scale bar = 5 µm; (Where zein (40%), tungsten (WO_3_) concentration- is 0.001% (WO_3_@zein1) and 0.008% (WO_3_@zein8)).

Proteins have secondary structures due to the presence of amide I and II groups which have distinguished vibrational peaks and so FT-IR provides information about this structure (Fig. 2)^43^. Neat-zein displays a considerable peak at 3,289 cm^-1^ referring to –NH stretching (from amide structure) and an association absorption peak of –OH that is formed by amino acid residues (i.e. adsorbed water on the surface) (Fig. 2a)^44^. It is possible to distinguish between 2 types of water: surface-entrapped water (our case) and hydrate (a crystal-structured water molecule). Zein is a hydrophobic material and insoluble in water, so surface water acts as a plasticizer for nanofibers. Those results are in harmony with previous results that showed that zein was significantly plasticized by water^45^. C–H stretching (asymmetric and symmetric) has strong peaks at 2876 cm^−1^, 2,949 cm^−1^, and 1446.2 cm^−1^ ^43^. Amide III (C–N), amide II (N–H), and amide I (C=O) stretching illustrate peaks at 1245.1 cm^−1^, 1,532 cm^−1^, and 1,644 cm^−1^, respectively^46^. Those results agree with the previous FT-IR analysis of neat zein^43,44^. FT-IR for WO_3_@Zein nanofiber scaffold shows unchanged peak positions and intensities (Fig. 2b,c). In other words, zein and WO_3_ are mixed physically without any chemical reaction. Those results are a line with the work of El Fawal et al. when adding WO_3_ to hydroxy ethyl cellulose (HEC) and there is no change in HEC peaks^37^.

#### X-ray diffraction (X-RD) analysis

**Figure 3:**
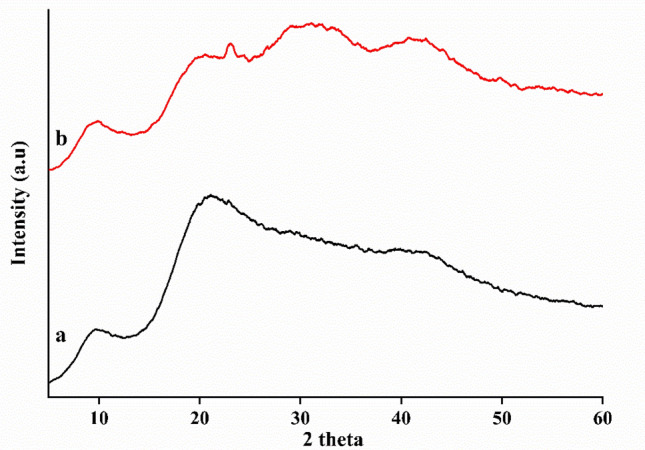
X ray Diffraction patterns of (**a**) neat zein and (**b**) WO_3_@zein8 nanofiber (Where Zein (40%), tungsten (WO_3_) concentration- is 0.008% (WO_3_@Zein8)).

X-RD data of neat zein and WO_3_@zein nanofibers scaffolds can be seen in Fig. 3. Neat zein nanofiber scaffold has an amorphous peak around 2θ = 9° and a wide peak around 2θ = 20°, as reported previously^47^. The first peak describes the α-helix structure of neat zein (intra-helix average backbone distance), while the second peak represents intra-helix packing between neighboring chains^48,49^. While, WO_3_ has two crystalline peaks at 2θ = 23.6°, and 31.8°, may be attributed to (0 0 2) and (2 0 0) planes (Reference pattern ICDD Card No. 00-043-0679) as earlier stated in the literature^36^. X-RD pattern of WO_3_@zein nanofiber scaffold shows coexistence of both peaks of WO_3_ and zein which are broad and less intense, which can be attributed to encapsulation of WO_3_ and its ability to form amorphous metal^50^. Also, crystalline peaks of WO_3_ disappeared in WO3@zein nanofiber scaffold may be due to the covering of crystalline form of WO_3_ by zein molecules. Those results are a line with Dhanalakshmi et al. when used ZnO Ns with starch molecule to prepare antibacterial film^51^.

#### Thermogravimetric analysis (TGA)

**Figure 4:**
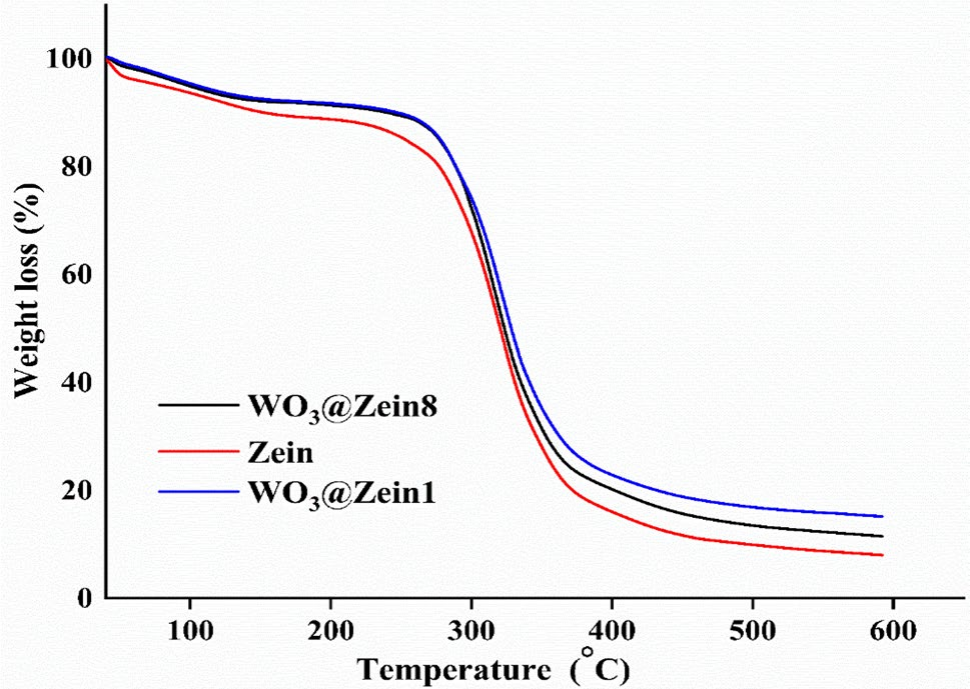
TGA analysis for the nanofiber (neat zein; WO_3_@zein1; WO_3_@zein8) (Where zein (40%), tungsten (WO_3_) concentration- is 0.001% (WO_3_@zein1) and 0.008% (WO_3_@zein8)).

Thermogravimetric curves of neat zein and WO_3_@zein nanofiber scaffolds are shown in Fig. 4. Thermal stability of prepared nanofibers scaffolds (neat zein and WO_3_@zein) is examined via thermal gravimetric analysis (TGA) by measuring mass loss as a function of temperature^52^. In all samples, the TGA curve has two weight loss stages. First stage (44.5–66.9 °C) refers to the loss of water and any residual solvent (acetic acid: ethanol) from the nanofiber^53^. Second stage (213–456 °C) relates to the progressive depolymerization and decarboxylation of thermally unstable protein units^54^. These results are in agreement with those obtained in previous TGA^54,55^. Furthermore, WO_3_ addition does not affect zein thermal stability due to the low concentration of WO_3_ compared to zein concentration. These results are consistent with those found when WO_3_ (low concentration) was added to hydroxyethyl cellulose (HEC) and HEC thermal stability remained unchanged^37^.

#### Cytotoxicity evaluation for nanofibers scaffolds

**Figure 5:**
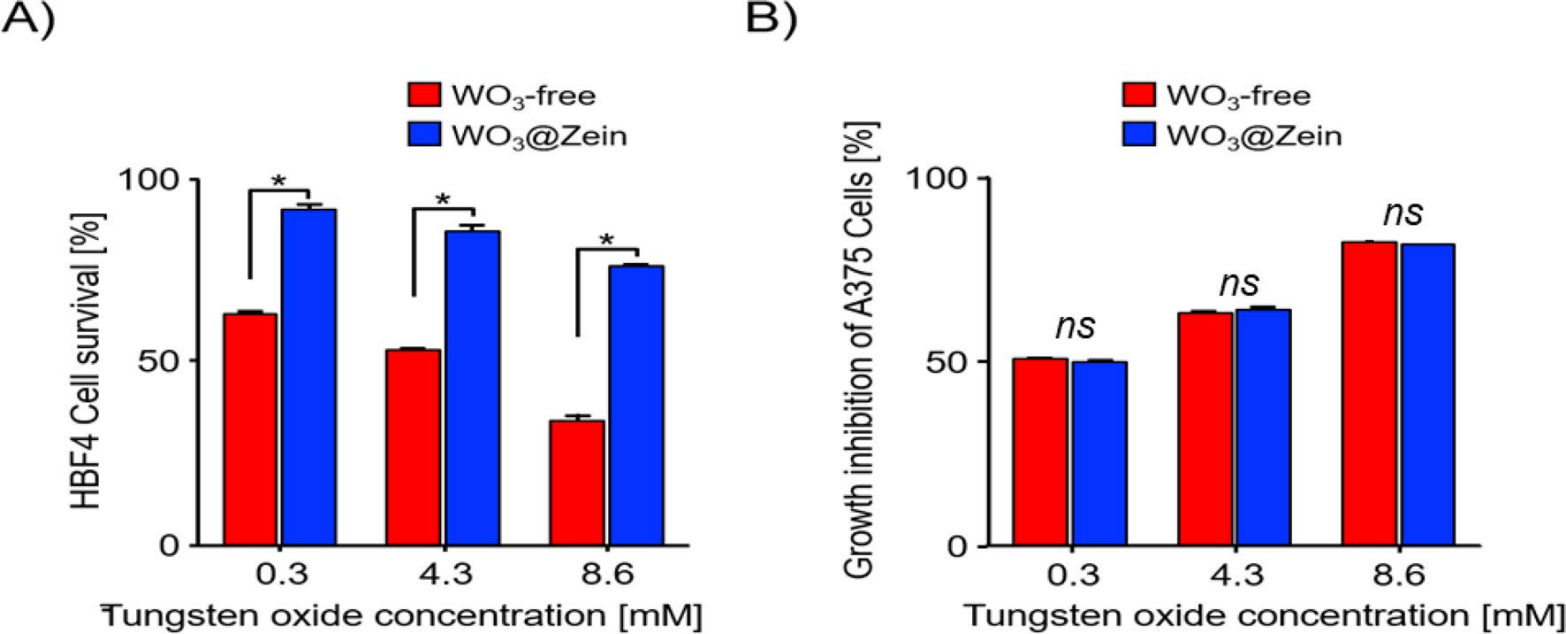
The cytotoxic effect of WO_3_ (WO_3_-free and WO_3_@zein nanofiber) on (**A**) human normal skin cells (HBF4), (**B**) human melanoma cells (A375) after 72 h incubation. Values are presented as mean ± SEM (n = 3). The comparisons are considered significantly different at *p* < 0.05* (ns = non-significant).

Cytotoxicity of WO_3_-free and WO_3_@zein nanofibers scaffolds is tested against HBF4 normal melanocytes and A375 cells to determine its potential toxicity on normal cells (Fig. 5). Results indicate that growth of the WO_3_- free-treated HBF4 cells (at 0.3, 4.3, and 8.6 mM) reduce to 63.09%, 53.41%, and 33.86%, respectively, compared to 91.64%, 85.68, and 76.14%, respectively, in the case of WO_3_@zein nanofiber scaffold. Therefore, the growth of WO_3_@zein-treated HBF4 cells is maintained at > 76% even at the highest dose (8.6 mM). These results confirm the ability of the newly developed formula to reduce the toxic effect of WO_3_ on normal cells. Tungsten was reported to accumulate in several organs and/or tissues such as kidneys, liver, ovaries, and uterus causing detrimental toxic effects which have limited its biomedical applications^56^. Limitations of metal/metal oxide toxicity, instability, and aggregation can be overcome through incorporation or encapsulation into polymers^57,58^. Polymers deliver metal/metal oxide with lower toxicity but with augmented efficiency when given at reduced doses ^46,59^. For instance, the cytotoxicity of WO_3_ (towards normal cells) was decreased when incorporated into hydroxyethylcellulose ^37^.

### Anticancer activity evaluation of nanofibers scaffolds

**Figure 6:**
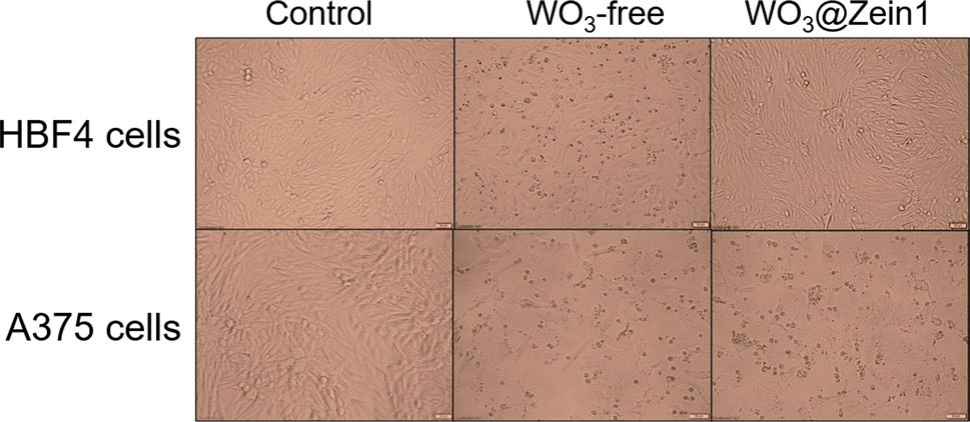
Morphological changes in human normal skin (HBF4) and melanoma (A375) cells after treatment with WO_3_ (0.3 mM) (WO_3_-free and WO_3_@zein nanofiber) for 72 h.

Figure 6 illustrates the anticancer potential test of WO_3_-free and WO_3_@zein nanofibers scaffolds at the same concentrations that were used in the cytotoxicity test. Results show that the nanofibers scaffolds sustain the growth inhibitory effect of WO_3_@zein nanofiber scaffolds on A375 melanoma cells (64.14%, 49.23%, and 81.73%) compared to the WO_3_-free form (63.18%, 50.86%, and 82.59%) at doses of 0.3, 4.3, and 8.6 mM, respectively. Also, the figure shows comparable damage in both normal and melanoma cell lines after 72 h treatment with WO_3_-free (0.3 mM). Both melanoma and normal cells are inhibited by 50.86% and 36.91%, respectively. In contrast, WO_3_@zein nanofiber scaffold (at the same concentration) exhibits only morphological damage in treated melanoma cells (which causes 50% growth inhibition) without having a significant effect on normal cell proliferation. The concentration of 0.3 mM of WO_3_ has been selected for further evaluation of the anticancer potential of the WO_3_@zein nanofiber scaffold. The pro-oxidant activity of tungsten oxide has been suggested that tungsten-containing compounds can act as catalysts for oxidation reactions^60^. Several studies have confirmed that tungsten oxide can elevate intracellular reactive oxygen species (ROS) and reduce the antioxidant capacity of mammalian cells^56,61^.

#### Measurement of the increment in intracellular ROS generation in A375 cells

**Figure 7:**
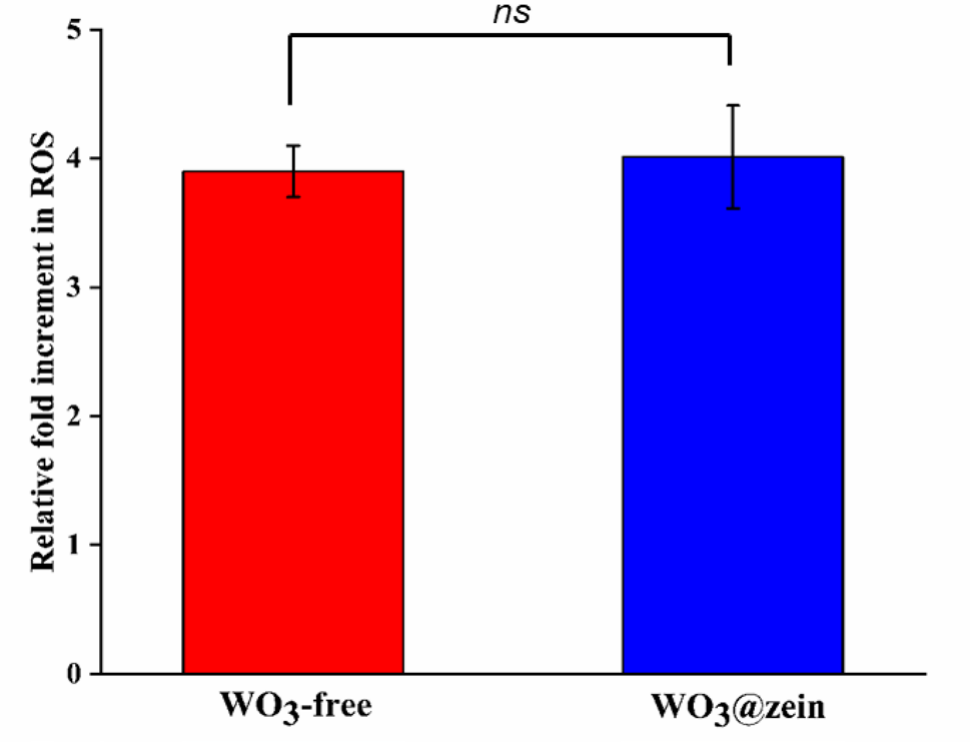
Reactive oxygen species (ROS) for melanoma cells (A375) after treatment by WO_3_ (0.3 mM) (WO_3_-free and WO_3_@zein nanofiber scaffold) for 72 h (values are presented as mean ± SEM (n = 3)) (ns = non-significant).

Reactive oxygen species (ROS) content of A375 cancer cells is evaluated after treatment with WO_3_-free and WO_3_@zein nanofiber scaffold (0.3 mM concentrations) in comparison to untreated control cells (Fig. 7). Results show that both WO_3_-free and WO_3_@zein nanofiber scaffold cause a fourfold increase in the cellular generation of ROS (3.94 ± 0.2 and 4.01 ± 0.4, respectively) relative to untreated cells. ROS are biomolecules with multiple functions in mammalian cells related to cellular signaling. Under normal cell conditions, elevated ROS levels induce oxidative stress, leading to cellular senescence, carcinogenesis, or cell death. Therefore, sustaining redox homeostasis in normal cells is crucial for cell survival^62^. On the contrary, metabolically demanding cancer cells have an elevated level of ROS due to uncontrolled proliferation^63^. However, a massive accumulation of ROS in cancer cells inhibits tumor growth by suppressing the proliferation signaling pathway, cell cycle, and the biosynthesis of nucleotides and ATP^64^. Therefore, finding pro-oxidant agents able to deprive cancer cells of the ability to control ROS levels is critical.

#### Quantitative detection for the change in the expression of proapoptotic genes and oncogenes in the treated cancer cells

**Figure 8:**
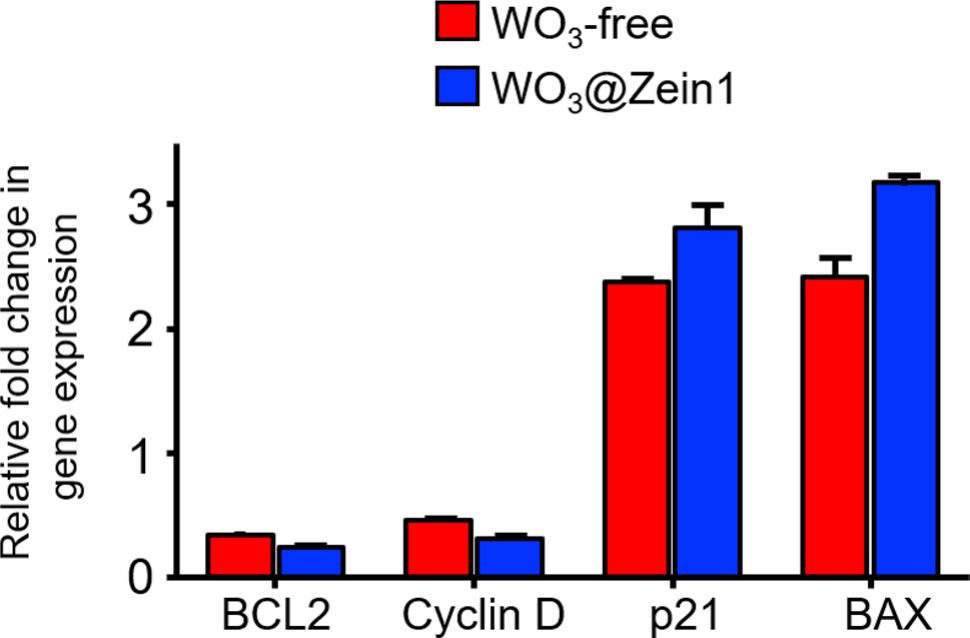
Relative fold change in the gene expression in melanoma cells (A375) after treatment by WO_3_ (0.3 mM) (WO_3_-free and WO_3_@zein nanofiber scaffold) for 72 h (values are presented as mean ± SEM (n = 3)).

Figure 8 presents a molecular study to elucidate the anticancer activity of WO_3_ (WO_3_-free and WO_3_@zein nanofiber scaffold) using qPCR. Oncogenes (B-cell lymphoma 2 “BcL-2” and cyclin D-mediated uncontrolled cell cycle) and for up-regulation of proapoptotic genes (cyclin-dependent kinase inhibitor p21 and Bcl-2-associated X protein “BAX”) are used as examples^65^. Treatment with WO_3_ (WO_3_-free and WO_3_@zein nanofiber scaffold) induced upregulation of p53-downstream genes (p21 and Bax) while downregulating oncogenes (BCL2 and cyclin D). Pro-oxidant activity of WO_3_-free and WO_3_@zein nanofiber scaffold can be linked to its suppressive effect on BCL2 and cyclin D through many explanations. For instance, Hildeman et al. reported that ROS could sensitize T cells to apoptosis by decreasing the expression of Bcl-2^66^. Similarly, BCL-2 inhibition in MIN6 mouse insuloma cells increased the production of peroxides^67^. Besides, exposure of HeLa human cervical cancer cells and HEK293 human embryo kidney cells to excessive ROS induced inhibition of cyclin D1. This was found to contribute to the induction of cell cycle arrest in G2 phase under oxidative stress^68^. The pro-oxidant activity of WO_3_-free and WO_3_@zein nanofibers scaffold is also linked to its promoting effect on the apoptosis-related genes p21 and Bax. For example, induction of apoptosis by p21 in sarcoma cell lines can be ameliorated with antioxidants and sarcoma cells undergoing p21-dependent cell death had higher sensitivity to oxidants^69^. Thioridazine induces Bax-dependent apoptosis by enhancing ROS production followed by ER stress^70^.

## Conclusion

In this paper, we have described an effective process to prepare biocompatible nanofiber scaffold from plant protein (zein) as anti-melanoma. Tungsten oxide (WO_3_) as a metal oxide was incorporated into zein nanofibers scaffold using electrospinning technique. Morphology and the thermal stability of zein nanofibers scaffold unchanged by WO_3_ addition. While, FT-IR and X-ray diffraction** (**X-RD) data confirmed the presence of WO_3_ in WO_3_@zein nanofiber scaffold. Cytotoxicity of WO_3_@zein nanofibers scaffolds confirmed its ability to reduce the toxic effect of WO_3_ on normal cells as the growth of HBF4 cells was maintained at > 76% even at the highest dose (8.6 mM). Moreover, anticancer results for WO_3_@zein nanofibers scaffolds exhibits only morphological damage in treated melanoma cells (which causes 50% growth inhibition) without having a significant effect on normal cell proliferation. The results demonstrate that WO_3_@zein nanofiber scaffold prepared by electrospinning could be used as a promising material candidate as anti-melanoma.

## Data Availability

The datasets used and/or analyzed during the current study available from the corresponding author on reasonable request.
